# International Collaboration to Save Children With Acute Lymphoblastic Leukemia

**DOI:** 10.1200/JGO.19.00010

**Published:** 2019-05-02

**Authors:** Ching-Hon Pui, Jing-Yan Tang, Jun J. Yang, Sai-Juan Chen, Zhu Chen

**Affiliations:** ^1^ St Jude Children’s Research Hospital, Memphis, TN; ^2^ Shanghai Children’s Medical Center, Shanghai Jiao Tong University, Shanghai, China; ^3^ Shanghai Institute of Hematology, Shanghai Jiao Tong University, Shanghai, China

Childhood acute lymphoblastic leukemia (ALL) is a modern era cancer success story that also is a paradigm for developing more effective treatment for all cancers. As a disease prototype that can be cured by pharmacotherapy alone, ALL is an ideal model for how national and international collaboration can help to cure more children worldwide.^1^ We have witnessed the life-saving power of such collaborations.

In 1991, Beijing Children’s Hospital and Shanghai Children’s Medical Center through Project HOPE developed a partnership program with St Jude Children’s Research Hospital. The initial focus was on education/training of physicians and nurses and provision of medical equipment/supplies to strengthen diagnostic capabilities. In 2003, the effort expanded to include improvement of access to treatment after the St Jude team learned that most families of children with ALL in China abandoned treatment for financial reasons; thus, only approximately 30% of the patients were treated.^2,3^

In response to the disparities in access to care and outcomes, St Jude established the Partner in Hope Foundation (Hong Kong), and developed a standardized, effective, and cost-efficient ALL treatment protocol with investigators of Shanghai Children’s Medical Center and Beijing Children’s Hospital in 2004. This protocol was open to underprivileged families of patients with low- or intermediate-risk ALL who sought treatment at either institution but could not afford it. The foundation funded the entire cost of treatment. The first patient was enrolled in the protocol in March 2005, and since, she has completed her education, become a teacher, married, and given birth to a healthy child. Three years into the protocol, multiple supportive care initiatives were launched. These included infection control, palliative care, and nursing education programs; data management; and housing for patients’ families during remission induction. Of the 152 patients treated, 128 (84%) are alive and in remission.

This outstanding clinical outcome and cost effectiveness of ALL therapy (< $11,000 [US] per patient) were reported in 2009^4^ and drew the attention of the Chinese Ministry of Health. At that time, the ministry was developing a major health care reform, the New Rural Cooperative Medical Scheme, in which central and local governments provide health insurance to citizens with catastrophic diseases. In 2010, childhood ALL, acute promyelocytic leukemia, and congenital heart disease were selected as the initial model diseases to test the new scheme.^5^ In the first year alone, access to treatment was provided to more than 7,000 children with low- or intermediate-risk ALL whose families could not afford therapy, which attests to the impact of this initiative. The insurance has since been extended to all children with ALL, regardless of their disease risk, and to those with other catastrophic diseases (eg, chronic renal failure, aplastic anemia).

Having helped to eliminate the financial barriers to treatment access for approximately 10,000 Chinese children annually, we launched ambitious new initiatives. The goal was to improve the quality of care and enhance survival rates of children with ALL. In 2014, Shanghai Children’s Medical Center undertook the first initiative and developed the China National ALL Study Group. Twenty major hospitals and medical centers that covered 65% of the Chinese population participated.^3^ The Children’s Cancer Group ALL-2015 protocol was developed on the basis of the St Jude Total Therapy XV study^6^ but modified per the treatment tolerance of Chinese patients. In 2014, the VIVA China Children’s Cancer Foundation was formed to support state-of-the-art minimal residual disease measurements, data management, internal monitoring, external auditing, and data safety monitoring. From November 2014 to September 2018, 5,225 patients were enrolled. The estimated 3-year patient survival rate is 93.3%.

Advances in leukemia control under these initiatives triggered several national-level activities. In 2017, the Chinese Ministry of Health approved the establishment of the National Children’s Medical Center, modeled after US National Cancer Institute–designated cancer centers, to pursue patient care, education, and research. In October 2018, a National Children’s Medical Center hematology-oncology alliance was created to promote clinical care and research by 49 tertiary hospitals in 27 provinces and municipalities, which covers 80% to 90% of the Chinese population. The Children’s Cancer Registry, the first comprehensive national pediatric leukemia cancer registry in China, also was initiated in October 2018 and has begun collecting demographic data, information on disease subtypes, and treatment outcomes for all children with newly diagnosed ALL prospectively and retrospectively to 2015. Nearly 600 hospitals across China are represented. The repository will enable the estimation of incidence and prevalence of childhood ALL and a more rigorous assessment of outcomes and close monitoring of the impact of various initiatives at the national level.

Biomedical discoveries are a predictable outcome of this collaboration. For example, recent work has identified new molecular subtypes (ie, *MEF2D*, *ZNF384*, *DUX4/ERG*, *ETV6-RUNX1*-like, *PAX5* P80R rearrangements) with prognostic and therapeutic implications in pediatric and adult ALL.^7,8^ With current cure rates for childhood ALL approaching 90% in many high-income countries,^9^ it has become increasingly difficult to study the remaining drug-resistant subtypes because of the small number of patients. Therefore, these carefully designed cooperative trials in China provide unique opportunities to address challenging questions that may affect efforts to improve ALL cure rates worldwide.

Finally, this international collaboration demonstrates that academic institutions, governmental and nongovernmental public health agencies, and advocacy groups can work together to dismantle the barriers to effective cancer care, even in a country as large and populous as China. The challenge now is to extend these advances globally. Initiatives such as the St Jude Global Program, its Global Alliance, and other efforts highlight the importance of incorporating a global vision into childhood cancer clinical and research programs. The WHO, St Jude, and many other stakeholders are working to achieve global response at a systems level. This work presents a unique opportunity to implement initiatives similar to the Chinese ALL program on a global scale.^10^
